# Anterior temporalis and masseter muscle activity with different centric relation locating methods: A randomized cross-over trial

**DOI:** 10.1038/s41598-025-21616-z

**Published:** 2025-10-13

**Authors:** Ahmed T. Nofal, John C. Kois, Reham Said Elbasty, A. A. Zaki

**Affiliations:** 1 Fixed Prosthodontics Department, Faculty of Dentistry, Cairo University, Cairo, Egypt; 2 Graduate Prosthodontics, Department of Restorative Dentistry, School of Dentistry, University of Washington, Seattle, WA US; 3 Private practice, Kois Center, Seattle, WA US; 4 School of Dentistry, Newgiza University, Giza, Egypt

**Keywords:** Centric relation, Muscle activity, Muscle deprogramming, Anterior bite planes, Occlusal devices., Occlusion, Prosthetic dentistry, Neurophysiology

## Abstract

The aim of the study was to evaluate anterior temporalis and masseter muscle activity and patient satisfaction after using the Leaf Gauge (LG), Lucia Jig (LJ), and Anterior Deprogrammer (AD) to locate centric relation (CR) in healthy adults. The research question was: “Which of the investigated CR locating methods will yield the lowest muscle activity, and the highest patient satisfaction score?”. Thirty healthy adults were randomly allocated to three sequences for implementing tested methods, with a 24-hour wash-out period. Surface electromyography (Myowise) measured muscle activity at rest (R), maximum intercuspal position (MIP), and after locating CR. Patient satisfaction was assessed using a visual analogue scale (VAS) and a questionnaire. Friedman’s test followed by Dunn’s test compared muscle activity and VAS scores between methods (α = 0.05). Inter-method comparison showed no significant differences in anterior temporalis activity. Intra-method comparison showed that CR muscle activity was lower than MIP but higher than rest for all methods (*P* < 0.001 for right, left, and bilateral means). For masseter muscle activity, AD showed significantly lower muscle activity compared to LG and LJ. LJ and AD activity at CR was comparable to rest and significantly lower than MIP. LJ was rated the most comfortable, while LG presented the least comfort (*P* = 0.018). AD and LJ effectively reduced muscle activity when used to locate CR. LJ is simple and comfortable for healthy adults, while LG has limited versatility. Low muscle activity while locating CR is essential, as elevated muscle activity might compress the articular disc and alter the position. clinicaltrials.gov Identifier: NCT06095856, registration date: 2023-10-11.

## Introduction

A consistent reference position of the jaw, known as the centric relation (CR), can be defined as an inter-arch relationship that is independent of tooth contact in which the condyles articulate in the anterior-superior position against the posterior slopes of the articular eminences; the mandible is limited to a purely rotary movement in this position. It is a repeatable, clinically useful position^1,2^. CR, being the stable musculoskeletal position of the condyles in the fossa, is one of the fundamental pillars of orthopedic stability, which in turn represents the primary objective of any rehabilitative treatment to ensure long-term success and reduce the risk of developing temporomandibular disorders (TMDs)^3^. Consequently, CR has been recommended to guide different procedures such as full mouth reconstruction, changing the occlusal vertical dimension, and treatment of occlusal diseases and TMDs^4^.

Multiple approaches have been reported for locating CR, that can be classified as operator-guided approaches, which include bimanual manipulation, Roth power bite, and chin-point guidance and patient-determined approaches, which include direct interocclusal records created in conjunction with an occlusal device, such as the Lucia jig (LJ), the leaf gauge (LG), and the Anterior deprogrammer (AD)^5–11^.

Operator-guided approaches have drawn criticism since it is possible for the operator to apply excessive force while locating CR, in addition to the inconsistency and variability of the amount of applied force^8–10^. It was also reported that excessive force used during such approaches is counteracted by a protective protrusive reflex of the mandible and elevated muscle activity^10^.

Patient-determined approaches allow the patient’s own muscle force to guide the condyles into CR, which may be advantageous because it is unlikely to exceed physiological boundaries, thus avoiding joint or muscle dysfunction. These approaches require posterior teeth separation before recording CR by using an occlusal device^8,9^.

This posterior separation reduces the activity of the elevator muscles and relaxes the lateral Pterygoids^12^ by blocking all proprioceptive impulses that create patient awareness of the jaw position in space, which guides mandibular closure in a position where maximum tooth contact exists (MIP), referred to as habitual contacts, thus preventing deviations and enabling an accurate recording of CR. Therefore, using an occlusal device to locate CR effectively reduces muscle activity and helps accurately locate CR without operator guidance or interference^13^.

In the process of locating CR, lower muscle activity must be guaranteed, as elevated muscle activity of the elevator muscles will compress the articular disc, which may compromise the position^14^.

Electromyography (EMG) studies were done to evaluate muscle activity of anterior temporalis and masseter muscles after locating CR using the leaf gauge^15,16^, the Lucia jig^13,15,17^, and the anterior deprogrammer^12^. Since the anterior deprogrammer is the most novel patient-oriented technique used for locating CR, literature is scarce regarding its effectiveness in reducing muscle activity compared with the previously established techniques. Additionally, patient satisfaction regarding various patient-oriented approaches has not been studied.

Therefore, the purpose of this cross-over clinical trial was to evaluate anterior temporalis and masseter muscle activity after using the leaf gauge, the Lucia jig and the anterior deprogrammer to locate CR in healthy adults, aiming to identify the method that yields the lowest muscle activity for both muscles (Primary Outcome). Furthermore, the study aimed to evaluate patient satisfaction after using the investigated methods (Secondary Outcome). The null hypotheses were:

- No difference in CR muscle activity would be found between the investigated methods.
- No difference in patient satisfaction would be found after locating CR with all investigated methods.

## Methods

All procedures were executed following the principles of the Helsinki Declaration and guided by the consolidated standards of reporting trials (CONSORT) extension for randomized crossover trials^18^. The research protocol was reviewed and approved by Faculty of Dentistry - Cairo University’s research ethics committee (approval number 1–7-23) and registered on clinicaltrials.gov (Identifier: NCT06095856, registration date: 2023-10-11).

PS software version 3.1.2 (https://ps-power-and-sample-size-calculation.software.informer.com/3.1/) was used to calculate the sample size. The alpha, beta probabilities, confidence level, and the study power were set to 0.05, 0.2, 95% and 80% respectively. Estimated mean difference, standard deviation, and effect size were calculated based on the muscle activity reported by Gaikwad et al.^12^ in their 2022 study.

Potential participants were recruited from Faculty of Dentistry in the period of November-December 2023. All potential participants received information about the study, filled the medical and dental history questionnaires, and were clinically examined. Additionally, all potential participants underwent a temporomandibular joint evaluation to confirm the absence of joint sounds or pain. The range of motion was assessed and confirmed to be within the normal limits (> 40 mm, maximum interincisal distance 53–58 mm), with no deviations or deflections during opening and closing, no muscle tenderness upon palpation, and negative load testing^19^.

Inclusion criteria were having a signed informed consent, and being between 20 and 60 years old^20^, ASA I or controlled ASA II^21^. Exclusion criteria were bone or joint diseases^22,23^, temporomandibular disorders, anterior cross bite, anterior open bite, any tooth mobility^8^, non-restored partial edentulism^24^, and parafunction^25,26^.

Intra-oral scans were made by a prosthodontist (A.T.N.) with 11 years of experience in digital dentistry for all eligible participants with an intra-oral scanner (Primescan Connect; Dentsply Sirona), following the clinical guidelines that have been reported to maximize the accuracy of intra-oral scans^27,28^. Before scanning the buccal bite, a leaf gauge (Huffman Numbered Leaf Gauge; Huffman Dental Products LLC) was used to establish a 1 mm separation between the closest maxillary and mandibular posterior teeth. The buccal bite was then recorded on both the right and left sides with the leaf gauge in place^11^. The digital scans were imported into a computer-aided design software (DentalCAD 3.0, Galway; exocad GmbH). The height of the anterior deprogrammer platform was adjusted to maintain the 1 mm separation between the closest posterior teeth, while its width was set at 3 mm. Horizontal flatness of the platform was verified both anteroposteriorly and laterally. The deprogrammer was then additively manufactured and post-processed according to the protocol reported by Revilla-León et al.^11^, it was then checked for fit and posterior teeth separation, but was not delivered to the participants.

Thirty participants were then randomly assigned to one of the three study groups by (R.S.B.) using an online random sequence generator (www.random.org). In this cross-over trial, the three investigated methods were tested on all eligible participants, blindly allocated to the three study groups using opaque sealed envelopes (allocation ratio 1:1:1). The only difference between the study groups was the sequence in which the investigated methods were tested^29^. The sequence of the interventions in the study groups was designed in a way so that each of the investigated methods was at the beginning of the sequence in one group, in the middle of the sequence in another group and at the end of the sequence in the last group. The study groups were named according to the sequence of the implemented interventions: LG-LJ-AD, LJ-AD-LG, AD-LG-LJ. A 24-hour wash-out period^29^ was employed between tested methods so that only one method per day was tested, resulting in a total of 3 consecutive days required to test the three methods for all participants in each study group, at roughly the same time of day for each participant^30,31^. Sample grouping and sequence of testing the investigated methods is shown in Table 1.

**Table 1:** Sample grouping.

Group	Day 1	Day 2	Day 3
LG-LJ-AD	leaf gauge	Lucia jig	Anterior deprogrammer
LJ-AD-LG	Lucia jig	Anterior deprogrammer	leaf gauge
AD-LG-LJ	Anterior deprogrammer	leaf gauge	Lucia jig

AD, anterior deprogrammer; LG, leaf gauge; LJ, Lucia Jig.

The patient was positioned in the dental chair with the chair back reclined at approximately 120^o^ so that the patient would be lying back in the chair midway between the upright and the supine positions^32–34^, and this position was set as a custom program on the dental unit for employing and testing all the investigated methods for all participants in the study. None of the participants reported tension or discomfort in that position. All procedures were done by one operator (A.T.N.).

Locating CR with the leaf gauge was done following the procedural steps described by Long^6^. Twenty leaves of a commercially available leaf gauge (Huffman Numbered Leaf gauge; Huffman Dental Products LLC) were placed between the upper and lower anterior teeth and participants were asked to bite gently on the gauge. The number of leaves was reduced one by one until participants felt the first contact between the posterior teeth. Then the number of leaves was increased by one, and the position was held until participants reported posterior tooth contact, then the process was repeated until participants could not feel any posterior tooth contact for a period of 10 min. At this point, CR was considered located.

**Fig. 1:**
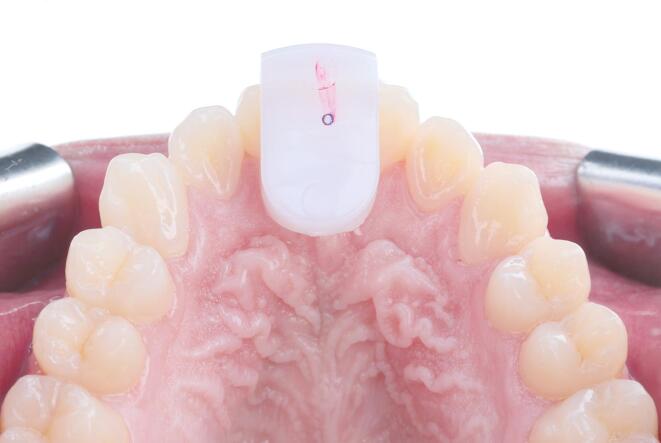
CR located with Lucia jig and verified by overlapping red and blue dots on retrusive end of trajectory marked on jig.

A standard procedure was adopted for locating CR with the Lucia jig, following the manufacturer’s instructions. Ready-made Lucia jigs (Lucia Jig; Great Lakes Dental Technologies) were painted with a small amount of silicone tray adhesive (Universal Tray Adhesive; Zhermack), then bite registration material (Occlufast Rock; Zhermack) was dispensed into the curved portion of the jig. The loaded jig was placed on the upper centrals and a Whale Tail (Whale Tails; Great Lake Dental Technologies) was placed directly beneath the jig to parallel the jig with the occlusal plane antero-posteriorly and side to side. Participants were asked to bite down and hold while the material was allowed to set for approximately 45 s. Once the material had set, the jig and the Whale Tail were removed, and the excess material was trimmed off with a No. 11 blade. The jig was then placed back on the participants’ upper central incisors. Using 40µ red articulating paper (Bausch Arti-Check Articulating Paper; Bausch GmbH & Co.), participants were asked to slide the lower incisors forward and back several times marking the jig, then the jig was removed, and the pattern of the lines was inspected to assure that the lines were on both edges or on the center of the jig. To determine the participants’ CR, the jig was replaced in the mouth, and participants were asked to bite down, slide forward, back, and then squeeze. This sequence was repeated twice and held after repeating. Then, participants were asked to open slightly, and a 40µ blue articulating paper was placed between the jig and the lower incisors. Participants were then asked to tap three times to mark the reference contact point of the lower incisors on the jig. This contact was verified using red articulating paper. The overlapping red and blue dots on the posterior end of the previously marked trajectory confirmed the reference position and CR was considered located. (Fig. 1)

**Fig. 2:**
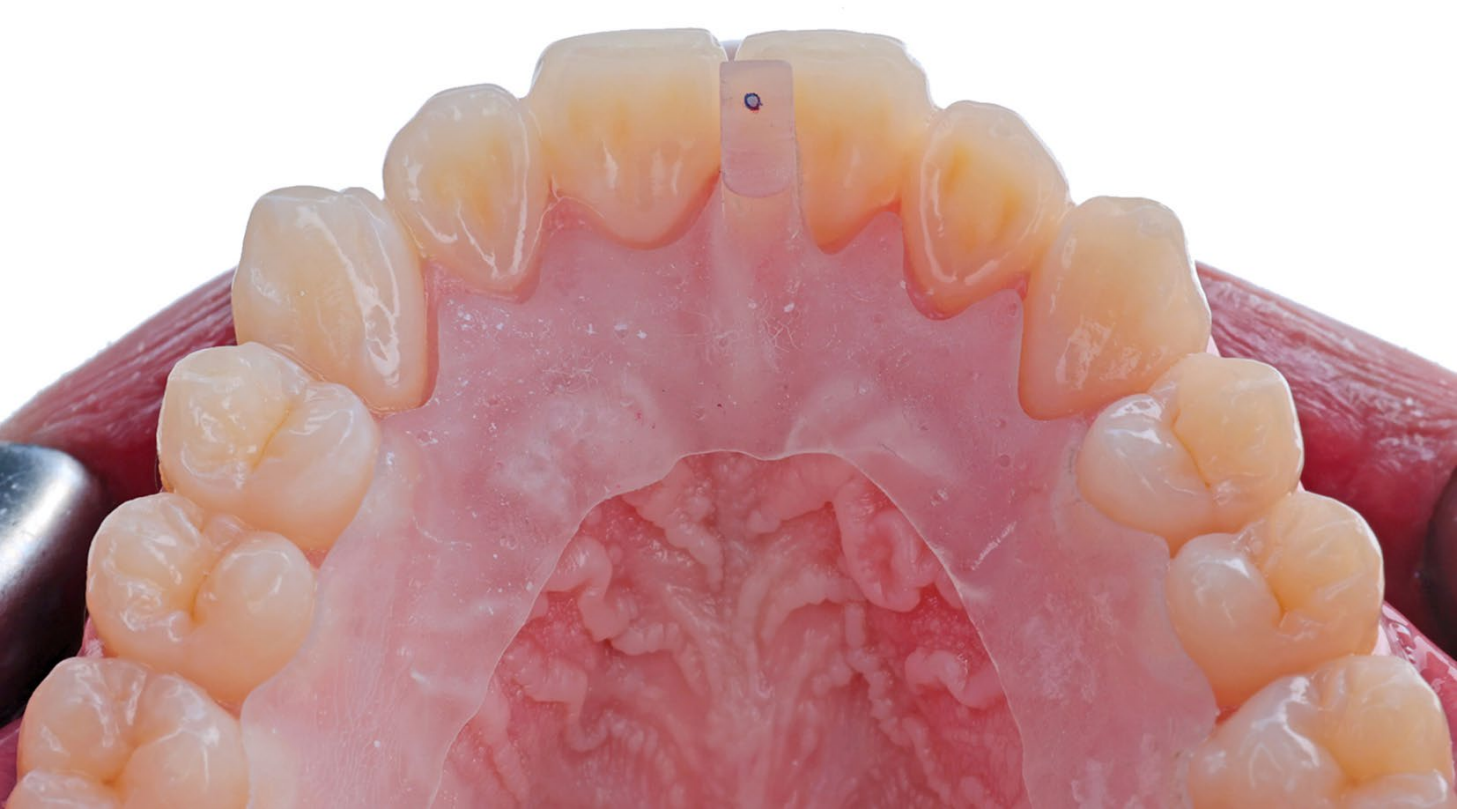
CR located using anterior deprogrammer and verified by overlapping red and blue dots on pre-adjusted platform.

When using the anterior deprogrammer, participants were asked to bite down, slide forward, back, and squeeze on the pre-adjusted anterior platform; the same sequence was repeated twice and held after repeating. Participants were then asked to open slightly while a blue articulating paper was placed between the platform and the lower central incisors. The participants were then asked to tap three times. The reference contact point of the lower incisors was marked in blue on the platform. Using red articulating paper, participants were asked to tap three more times. Overlapping red and blue dots (Fig. 2) confirmed the reference position and CR was considered located^11^.

**Fig. 3:**
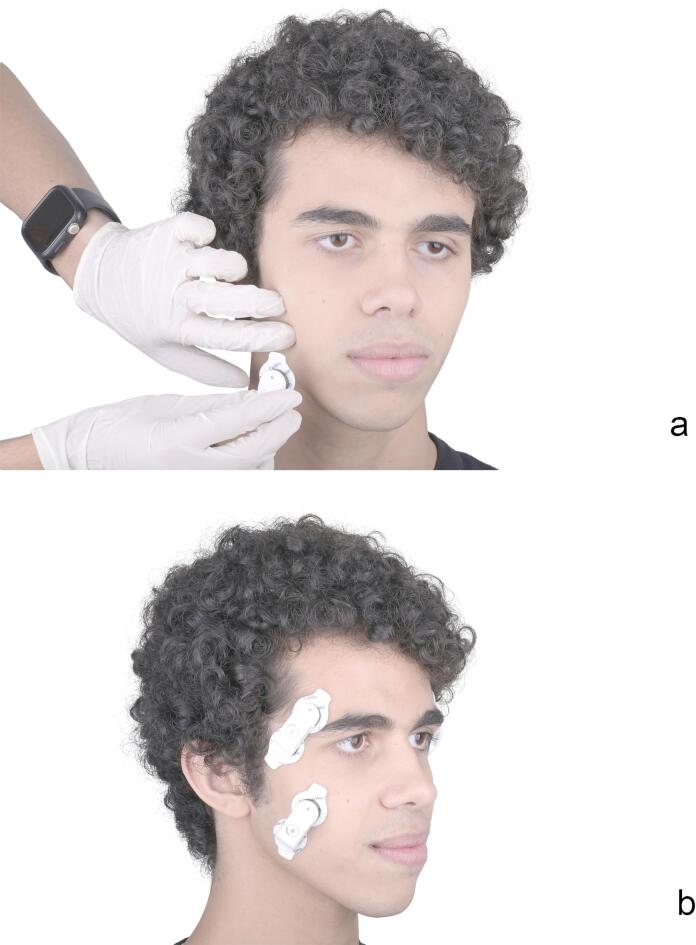
Attachment of sEMG transducers. a, Muscle palpation before attaching transducer. b, Transducers attached on anterior temporalis and masseter muscles.

sEMG^35,36^ readings were recorded using wireless transducers (Myowise; Cometa Systems). The area of the skin at the site of measurement was shaved if needed, cleaned, then rubbed with ethyl alcohol to reduce the skin impedance^12,13^. One transducer was attached to each muscle on both sides strictly abiding by the SENIAM guidelines^37^, participants were asked to tap their teeth repeatedly to locate the motor point of each muscle. The transducers were then positioned on the identified motor point, aligned with the direction of the muscle fibers. (Fig. 3a and b) Muscle activity was recorded three times for each participant on each day, first in the physiologic rest position (R) of the mandible by asking the participants to relax all their facial muscles and just breathe through their mouths^19^, then in maximum intercuspal position (MIP) by asking the participants to bite gently so that all their teeth are touching lightly, and lastly, in CR once it had been located using any of the investigated methods. Analysis was performed by a single-blinded trained professional using an EMG analysis software (EMG & Motion Tools v8.7; Cometa Systems) to provide a root mean square reading in microvolt (µV) for each muscle.

Patient satisfaction was recorded using a visual analog scale (VAS) on the third day after using the last investigated method. Additionally, a questionnaire was used to better interpret the VAS score. Questions for each method were: 1- Was the method used comfortable? 2- Were the doctor’s instructions easy to follow? 3- Was there any pain of any kind while this method was being used?

Statistical analysis was performed with a statistical software program (IBM SPSS Statist﻿ics for Windows, v23.0; IBM Corp). Quantitative data were explored for normality by checking the distribution of data and using tests of normality (Kolmogorov-Smirnov and Shapiro-Wilk tests). The significance level was set at *P* ≤ 0.05.

## Results

All data showed non-normal (non-parametric) distribution. Quantitative data were presented as median, range, mean and standard deviation (SD) values. Friedman’s test followed by Dunn’s test were used to compare the three methods. Wilcoxon signed-rank test was used to compare between R, MIP, and CR positions. Cochran’s Q test was used to compare satisfaction questions.

**Fig. 4:**
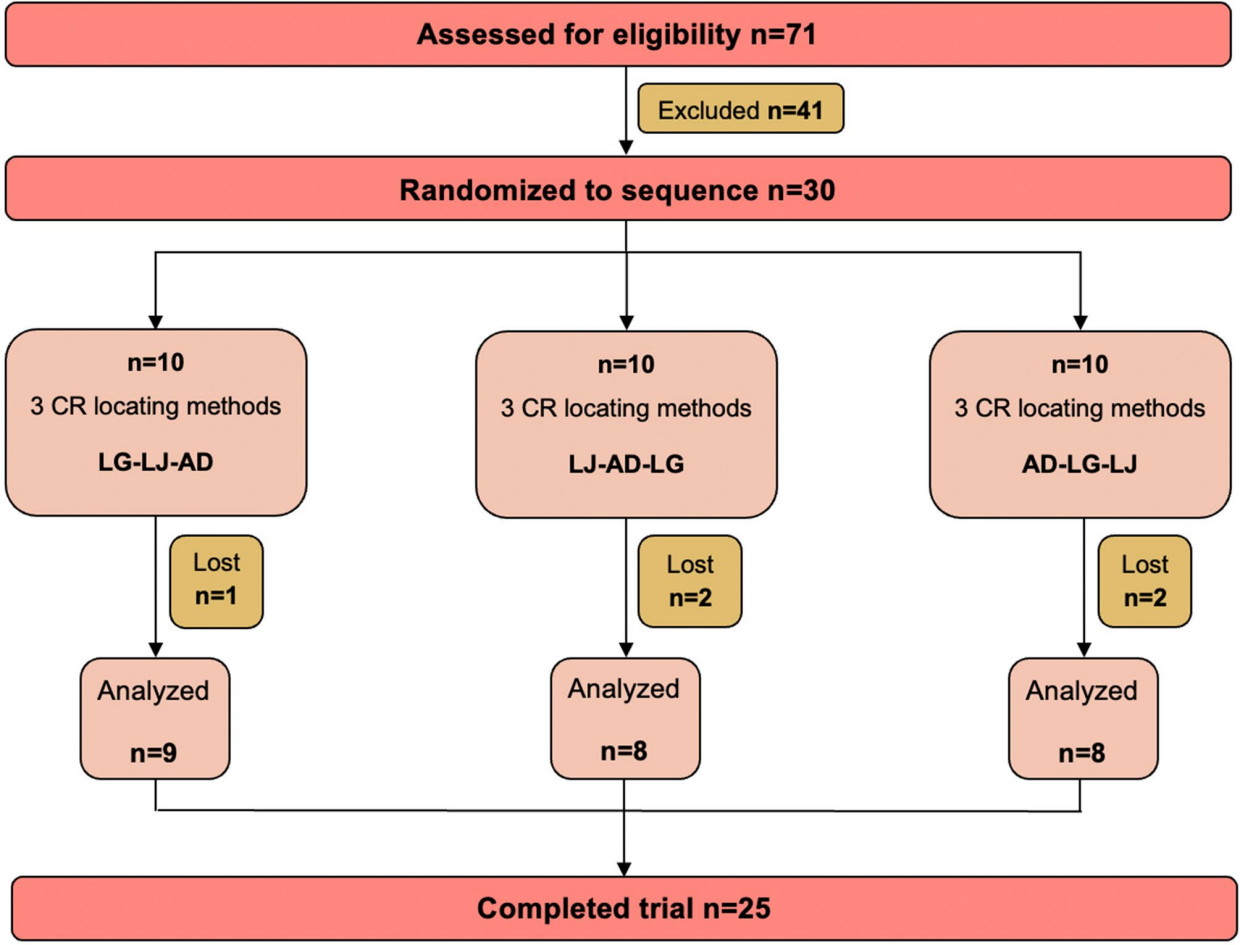
CONSORT flow diagram.

The CONSORT flow diagram^38^ (Fig. 4) shows that, out of the 71 potential participants who volunteered for the trial, only 30 were eligible for enrollment in the study. One participant in group LG-LJ-AD, 2 participants in group LJ-AD-LG and 2 participants in group AD-LG-LJ were excluded from the study as they were absent on at least one of the testing days. The remaining 25 participants (13 males [52%] and 12 females [48%]; age range: 22–56) had a mean age and standard deviation of 39 (9.4) as shown in Table 2.

**Table 2:** Descriptive statistics for baseline characteristics.

Gender	Male (*n* = 13)	Female (*n* = 12)	Total (*n* = 25)
Age [Mean, (SD)]	36.7 (7.7)	41.6 (10.7)	39 (9.4)

Regarding muscle activity of the anterior temporalis muscle, comparison of CR muscle activity between the three methods (Inter-method comparison) revealed that whether the right (*P* = 0.882) or left (*P* = 0.607) sides or the mean of both sides (*P* = 0.646) were measured, there was no statistically significant difference between the three methods as shown in Table 3. The Intra-method comparison between R, MIP and CR muscle activity for each method is reported in Table 4, and shows that for the leaf gauge (*P* < 0.001 for Rt, Lt, and mean of both sides), the Lucia jig (*P* < 0.001 for Rt, Lt, and mean of both sides), and the anterior deprogrammer (*P* < 0.001 for Rt, Lt, mean of both sides), a statistically significant difference was observed between R, MIP, and CR. Pair-wise comparisons revealed that MIP showed the statistically significant highest muscle activity. CR showed statistically significant lower muscle activity. Rest showed the statistically significant lowest muscle activity.

**Table 3:** Descriptive statistics and results of Friedman’s test for comparison between centric relation muscle activity (µV) of anterior temporalis muscle as assessed by three methods.

Side	Leaf gauge		Lucia jig		Anterior deprogrammer		*P*	Effect size	
	Median (Range)	Mean (SD)	Median (Range)	Mean (SD)	Median (Range)	Mean (SD)			
Right	6.3 (3.8, 9.8)	6.6 (1.8)	6 (4, 14.5)	6.6 (2.3)	5.9 (3.7, 14.6)	6.6 (2.5)	0.882		0.005
Left	6.2 (3.8, 14)	6.5 (2.3)	6.2 (4.6, 12.1)	6.9 (2.1)	6.1 (3.5, 13.8)	6.9 (2.7)	0.607		0.021
Mean of both sides	6.2 (3.8, 14)	6.6 (2.1)	6.1 (4, 14.5)	6.8 (2.2)	5.9 (3.5, 14.6)	6.7 (2.5)	0.646		0.018

*Significant at *P* ≤ 0.05.

**Table 4:** Descriptive statistics and results of Friedman’s test for comparison between anterior temporalis muscle activities (µV) at rest, maximum intercuspal position (MIP), and centric relation (CR).

Method	Side	Rest		MIP		CR		*P*	Effect size
		Median (Range)	Mean (SD)	Median (Range)	Mean (SD)	Median (Range)	Mean (SD)		
Leaf gauge	Right	4.8 (3.4, 6.9) ^C^	4.8 (0.9)	8.1 (4.6, 16.2) ^A^	8.4 (2.6)	6.3 (3.8, 9.8) ^B^	6.6 (1.8)	< 0.001*	0.622
	Left	5.3 (3.4, 7.2) ^C^	5.2 (1)	7.7 (4.6, 28.8) ^A^	9.8 (4.9)	6.2 (3.8, 14) ^B^	6.5 (2.3)	< 0.001*	0.601
	Mean of both sides	5.1 (3.4, 7.2) ^C^	5 (1)	8 (4.6, 28.8) ^A^	9.1 (4)	6.2 (3.8, 14) ^B^	6.6 (2.1)	< 0.001*	0.611
Lucia jig	Right	4.5 (3.5, 7.2) ^C^	4.6 (0.7)	7.4 (3.7, 15.2) ^A^	7.8 (2.4)	6 (4, 14.5) ^B^	6.6 (2.3)	< 0.001*	0.658
	Left	5 (3.8, 7.8) ^C^	5.2 (0.9)	7.9 (4.8, 15) ^A^	8.5 (3)	6.2 (4.6, 12.1) ^B^	6.9 (2.1)	< 0.001*	0.502
	Mean of both sides	4.7 (3.5, 7.8) ^C^	4.9 (0.8)	7.5 (3.7, 15.2) ^A^	8.1 (2.7)	6.1 (4, 14.5) ^B^	6.8 (2.2)	< 0.001*	0.593
Anterior deprogrammer	Right	4.3 (3.1, 6.6) ^C^	4.5 (0.9)	6.9 (4, 16.7) ^A^	7.6 (3.3)	5.9 (3.7, 14.6) ^B^	6.6 (2.5)	< 0.001*	0.516
	Left	4.2 (3.5, 7) ^C^	4.7 (1)	6.6 (4, 15.5) ^A^	7.7 (3.2)	6.1 (3.5, 13.8) ^B^	6.9 (2.7)	< 0.001*	0.426
	Mean of both sides	4.3 (3.1, 7) ^C^	4.6 (1)	6.7 (4, 16.7) ^A^	7.7 (3.3)	5.9 (3.5, 14.6) ^B^	6.7 (2.5)	< 0.001*	0.471

*Significant at *P* ≤ 0.05, different superscripts in same row indicate statistically significant difference between positions.

Comparison of CR masseter muscle activity between the three methods (Inter-method comparison) revealed that whether at the right (*P* = 0.037) or left sides (*P* = 0.023) or the mean of both sides (*P* = 0.001), there was a statistically significant difference between the three methods. Pair-wise comparisons revealed that there was no statistically significant difference between the Leaf gauge and Lucia jig methods; both showed statistically significant higher muscle activity than the anterior deprogrammer as shown in Table 5. Table 6 shows the comparison between R, MIP and CR muscle activity for each method (Intra-method comparison). A statistically significant difference was noted between R, MIP, and CR for the leaf gauge on the right (*P* < 0.001) and left sides (*P* < 0.001). Pair-wise comparisons revealed these statistically significant differences: MIP showed the highest muscle activity, CR showed lower muscle activity, and Rest showed the lowest muscle activity. As for the mean of both sides, there was a statistically significant difference (*P* < 0.001) between Rest, MIP, and CR. Pair-wise comparisons revealed that MIP showed the statistically significant highest muscle activity. There was no statistically significant difference between CR and R position; both showed a statistically significant lower muscle activity. A statistically significant difference was found on the right and left sides, as well as the mean of both sides between R, MIP, and CR for both the Lucia Jig (*P* < 0.001) and the anterior deprogrammer (*P* < 0.001). Pair-wise comparisons revealed that MIP showed the statistically significant highest muscle activity. There was no statistically significant difference between CR and Rest positions; both showed the statistically significant lowest muscle activities.

**Table 5:** Descriptive statistics and results of Friedman’s test for comparison between centric relation muscle activity (µV) of masseter muscle as assessed by three methods.

Side	Leaf gauge		Lucia jig		Anterior deprogrammer		*P*	Effect size
	Median (Range)	Mean (SD)	Median (Range)	Mean (SD)	Median (Range)	Mean (SD)		
Right	5.6 (3.7, 23.5) ^A^	7 (4.4)	5 (3.2, 17.2) ^A^	6.2 (3.4)	4.1 (3.3, 9.3) ^B^	4.8 (1.6)	0.037*	0.137
Left	7.8 (6.3, 25.1) ^A^	8.9 (3.8)	7.3 (5.9, 13) ^A^	7.6 (1.4)	6.7 (6.2, 13.6) ^B^	7.3 (1.7)	0.023*	0.158
Mean of both sides	6.8 (3.7, 25.1) ^A^	8 (4.2)	6.7 (3.2, 17.2) ^A^	6.9 (2.7)	6.3 (3.3, 13.6) ^B^	6.1 (2.1)	0.001*	0.213

*Significant at *P* ≤ 0.05, different superscripts in same row indicate statistically significant difference between methods.

**Table 6:** Descriptive statistics and results of Friedman’s test for comparison between masseter muscle activities (µV) at Rest, maximum intercuspal position (MIP), and centric relation (CR).

Method	Side	Rest		MIP		CR		*P*	Effect size
		Median (Range)	Mean (SD)	Median (Range)	Mean (SD)	Median (Range)	Mean (SD)		
Leaf gauge	Right	4.4 (3.5, 15.6) ^C^	5 (2.6)	8 (4.2, 17.5) ^A^	8.5 (4)	5.6 (3.7, 23.5) ^B^	7 (4.4)	< 0.001*	0.416
	Left	6.6 (6.1, 10.4) ^C^	7.1 (1.1)	9.2 (6.3, 14.1) ^A^	9.4 (2.4)	7.8 (6.3, 25.1) ^B^	8.9 (3.8)	< 0.001*	0.378
	Mean of both sides	6.2 (3.5, 15.6) ^B^	6 (2.2)	8.4 (4.2, 17.5) ^A^	9 (3.3)	6.8 (3.7, 25.1) ^B^	8 (4.2)	< 0.001*	0.264
Lucia jig	Right	4.4 (3.5, 12.5) ^B^	4.9 (1.9)	7 (4.2, 24.5) ^A^	9.2 (5.8)	5 (3.2, 17.2) ^B^	6.2 (3.4)	< 0.001*	0.455
	Left	6.9 (5.9, 11.8) ^B^	7.3 (1.3)	8.9 (6.1, 19.2) ^A^	9.8 (3)	7.3 (5.9, 13) ^B^	7.6 (1.4)	< 0.001*	0.418
	Mean of both sides	6.1 (3.5, 12.5) ^B^	6.1 (2)	8.2 (4.2, 24.5) ^A^	9.5 (4.6)	6.7 (3.2, 17.2) ^B^	6.9 (2.7)	< 0.001*	0.271
Anterior deprogrammer	Right	4.1 (3.5, 9.2) ^B^	4.6 (1.3)	6.4 (3.7, 21.6) ^A^	8.5 (4.7)	4.1 (3.3, 9.3) ^B^	4.8 (1.6)	< 0.001*	0.453
	Left	6.4 (5.8, 11.2) ^B^	6.9 (1.4)	10.1 (6.5, 16.6) ^A^	10.1 (2.8)	6.7 (6.2, 13.6) ^B^	7.3 (1.7)	< 0.001*	0.593
	Mean of both sides	6.1 (3.5, 11.2) ^B^	5.8 (1.7)	9.1 (3.7, 21.6) ^A^	9.3 (3.9)	6.3 (3.3, 13.6) ^B^	6.1 (2.1)	< 0.001*	0.336

*Significant at *P* ≤ 0.05, different superscripts in same row indicate statistically significant difference between positions.

Regarding the patient satisfaction scores, there was a statistically significant difference (*P* = 0.018) between the three methods. Pair-wise comparisons between methods (Table 7) revealed no statistically significant difference between Lucia jig and anterior deprogrammer; both showed statistically significant higher satisfaction scores than Leaf gauge.

**Table 7:** Descriptive statistics and results of Friedman’s test for comparison between patient satisfaction scores for three methods.

Leaf gauge		Lucia jig		Anterior deprogrammer		*P*	Effect size	
Median (Range)	Mean (SD)	Median (Range)	Mean (SD)	Median (Range)	Mean (SD)			
8 (2, 10) ^B^	7.3 (2.4)	10 (5, 10) ^A^	8.9 (1.5)	10 (4, 10) ^A^	8.8 (1.9)	0.018*		0.161

*Significant at *P* ≤ 0.05, different superscripts indicate statistically significant difference between methods.

There was a statistically significant difference between participants’ answers to the comfort question (Table 8). The most comfortable method was the Lucia jig (*P* = 0.018) followed by the anterior deprogrammer while the Leaf gauge presented the least comfort. No statistically significant difference was found with ease of instructions (*P* = 0.368) or the presence of pain (*P* = 0.311) among all methods.

**Table 8:** Descriptive statistics and results of Cochran’s Q test for comparison between participants’ answers to questionnaire.

Question	Leaf gauge		Lucia jig		Anterior deprogrammer		*P*
	*n*	%	*n*	%	*n*	%	
Comfort	19	76	25	100	23	92	0.018*
Ease of instructions	24	96	25	100	25	100	0.368
Presence of pain	4	16	1	4	2	8	0.311

*Significant at *P* ≤ 0.05.

## Discussion

To the best of the authors’ knowledge, the only study that investigated muscle activity following the use of an anterior deprogrammer, among other methods, was conducted by Gaikwad et al.^12^. However, their study had several limitations, including issues related to the CR locating posture, deprogramming procedures, and the wash-out period, all of which may have influenced the muscle activity measurements while locating CR. These limitations were addressed in the present study.

It has been shown that with posterior teeth separation, the proprioceptive reflexes are blocked and elevator muscle activity is reduced, which allows various patient-determined methods to locate the condyles in CR without operator guidance or interference^13^. Another factor to be considered while locating CR is that higher values of muscle activity can alter the position, as compression of the articular disc occurs, leading to a “forced position”^14^. Consequently, it can be assumed that the ideal patient-oriented method will be the most effective in reducing elevator muscle activity while locating CR, and therefore, the aim of the inter-method comparison was to measure the CR muscle activity and compare that activity between the three methods tested (LG, LJ, AD).

While some authors use the terms “deactivation” of elevator muscles^12^, and others use the term elevator muscle “shutdown”^19^, it is believed that joint stability is achieved through constant mild contraction of the elevator muscles at rest, known as muscle tonus. Absence of muscle tonus will result in separation of the articular surfaces and joint instability^19^. Therefore, the intra-method comparison explored the effectiveness of the 3 investigated methods in reducing CR muscle activity from that of MIP and evaluating the discrepancy between CR and resting muscle activity.

After locating CR using the investigated methods, no CR record was made, instead, muscle activity was recorded immediately after CR was considered located. This approach was chosen because the aim of the study was to assess muscle activity after the process of locating CR, not after its recording. Moreover, placing a CR record in situ would have altered the sEMG values, as participants would be actively biting on the record, thereby affecting the muscle activity being measured.

Masticatory muscles electromyography can be done using needle electrodes^39^ or surface electrodes^36^. Needle electrodes are more precise but more invasive, which may present some drawbacks for the study participants^35^, while sEMG utilizes non-invasive electrodes placed on the skin. In addition to its widespread use for evaluating temporalis and masseter muscle activity, sEMG provides ease of application, therefore, sEMG was the method of choice for outcome measurement in this study.

The inter-method comparison showed no significant difference in muscle activity for the anterior temporalis muscle, but the CR masseter muscle activity when using the Anterior deprogrammer was significantly lower than that of either the Lucia jig or the leaf gauge. Therefore, the first null hypothesis was accepted for the anterior temporalis muscles and rejected for the masseter muscles.

Inter-method comparison for the anterior temporalis muscles revealed that the CR muscle activity of the anterior deprogrammer was not statistically significantly different from that of the Lucia jig or the leaf gauge regardless of the location measured, this may imply that the different designs of the investigated devices did alter the neuromuscular demand on the anterior temporalis while locating CR. This was in contrast with the findings of three previously published studies. Gaikwad et al.^12^ reported that the temporalis muscle activity in CR when using the anterior deprogrammer was significantly less than that of the leaf gauge or the Lucia jig. This might be due to the different study timeline and wash-out period of the Gaikwad et al. study. Hickman et al.^40^ and Hickman and Cramer^41^ concluded that the leaf gauge resulted in the lowest CR muscle activity, which may be explained by the difference of the comparators between the present study and those studies.

Inter-method comparison for the masseter muscles revealed that the CR muscle activity of the anterior deprogrammer was significantly lower than that of the Lucia jig and the leaf gauge, this can be attributed to the ease of intra-oral retention of the anterior deprogrammer; the patient does not need to exert any pressure to keep the device in place. This finding was in accordance with the findings of Gaikwad et al.^12^, which may be due to the similar design of the anterior deprogrammer between both studies.

Intra-method comparison for the anterior temporalis and masseter muscles revealed that, for all methods, whether at the right, left sides, or the mean of both sides, muscle activity in CR was significantly lower than when the patient was in MIP. This may be because, despite different in design, all investigated devices distributed forces evenly in CR, enough to prevent excessive muscle activation. This was in agreement with the findings of Becker et al.^17^ and Santosa et al.^15^, who concluded that the leaf gauge and the Lucia jig significantly decreased muscle activity for all muscles. The similarity in findings may be attributed to the use of comparable CR locating devices and techniques. Intra-method comparison for the anterior temporalis muscles also revealed that, whether at the right, left sides, or the mean of both sides, CR muscle activity was significantly higher than that of the resting muscle activity. This can be supported by the findings of Williamson et al.^42^, Nassar et al.^13^, and Gaikwad et al.^12^, who reported that the temporalis muscle activity may be elevated because they are actively engaged in seating the condyles while locating CR with a patient-determined method. This may explain the higher anterior temporalis muscle activity observed in CR compared to R, regardless of the device used.

Intra-method comparison for the masseter muscles revealed that the leaf gauge’s CR muscle activity, whether at the right or the left sides, was significantly higher than the resting muscle activity, which can be attributed to the design of the leaf gauge, as it has no means of intra-oral retention, therefore it must be maintained in position by elevator muscle contraction. Regarding the mean of both sides, CR muscle activity with the leaf gauge was higher than the resting muscle activity, however, it’s worth noting that the difference was not statistically significant. It is also worth noting that no surrogate endpoint can be reached when CR is located using the leaf gauge, as CR is considered located after 10 min of no tooth contact with the leaf gauge in place. This differs from the Lucia jig and the anterior deprogrammer, where validation of the condylar position on the rotational axis of CR can be easily performed by checking the repeatability of the contact (overlapping red and blue dots) on the flat bite plane of both devices as shown in Figs. 1 and 2. Still, if proper patient selection was done, and gentle pressure was applied by the patient on the leaf gauge^8^, it can be a valid and reliable tool for locating CR and reducing muscle activity, which was the case in the present study^8,15,17^.

Intra-method comparison for the masseter muscles also revealed that the CR muscle activity of the Lucia jig and the anterior deprogrammer showed no statistically significant difference when compared with their respective muscle activity at rest, whether at the right, left or the mean of both sides. This finding highlights the effectiveness, validity and reliability of the Lucia Jig and the anterior deprogrammer in reducing the muscle activity while locating CR. This can be explained by the horizontally flat bite plane that allows passive placement of the mandible in CR^9^, unlike the leaf gauge, which can be considered a restrictive device, since forward movement of the condyles is not possible while using the leaf gauge. Since the leaves are placed between the palatal surfaces of the upper anterior teeth and the labial surfaces of the lower anterior teeth, a leaf gauge would be contraindicated in patients with constricted chewing pattern^8^. Additionally, if the use of the leaf gauge is combined with strong elevator muscle contraction and/or a deep overbite, the leaf gauge tends to push the condyles distally and often functions as a directive device^8^.

Literature classifies the Lucia jig and the leaf gauge as anterior deprogrammers^16^. The authors’ opinion is that the Lucia jig and the leaf gauge only provide posterior separation^8,9^, which deactivates the proprioceptive reflexes responsible for the mandibular closure engram (muscle memory)^43^ that guides the mandible into MIP’s habitual contacts^13,16,44^. On the other hand, muscle deprogramming involves the neuroplastic ability of the brain stem and the central pattern generator to formulate new synaptic pathways for an altered mandibular closure engram^45^ based on the altered environmental input, not just deactivating the pre-existing engram. In other words, the brain is re-learning the ability to guide the mandible into closure, which can occur through neurologic processes like long-term potentiation (LTP) and long-term depression (LTD)^46^. If an occlusal device is to be classified as a deprogrammer, its design must provide comfort and ease of intra-oral retention, protection against super-eruption, allow control over the amount of arch separation, and not hinder speech or normal tongue movements^12^. This is to allow for individual variations vis-à-vis the time required for the formulation of an altered closing engram^47^, which can occur in as fast as two minutes^43^, or longer depending on how fast the brain can learn^47^, or does not occur at all^45^. The anterior deprogrammer was effective in reducing the muscle activity of both anterior temporalis and masseter muscles compared to the other methods. Additionally, it fulfills all the requirements listed earlier for a device to be classified as a deprogrammer. It is also worth mentioning that for jaw stabilization or long-term deprogramming, a full-coverage occlusal stabilizing splint is the device of choice^48,49^. On the other hand, the use of the Lucia jig and the leaf gauge for prolonged periods of time is impractical^12^, and some authors negated their effect in breaking muscle engrams^8,9,16^. Furthermore, the prolonged use of short-term CR locating devices is contraindicated, as it may contribute to occlusal and joint disorders. They are also unsuitable for patients with TMDs who present a positive load test, indicating intra-capsular derangement, since their use may further destabilize the joint^19^.

The authors are unaware of any previous study that evaluated patient satisfaction regarding various methods used to locate centric relation in healthy adults. Based on the statistical analysis of the VAS and the questionnaire answers, the second null hypothesis was rejected.

Questionnaires were filled on the third day, after all investigated methods had been used by each participant. Handing the questionnaire after only one or two methods might have introduced bias, as earlier methods would have fewer or no comparators.

Statistical analysis of the VAS showed no statistical significance between the scores of the Lucia jig and the anterior deprogrammer, both showing significantly higher satisfaction scores than the leaf gauge. This may be due to time spent locating CR with the leaf gauge, which was higher than that required to locate CR using the Lucia jig or the anterior deprogrammer.

Regarding the questionnaire, the Lucia jig was statistically significantly the most comfortable method followed by the anterior deprogrammer, while the leaf gauge presented the least comfortable method. This can be due to the simplicity of the design of the Lucia jig, while the palatal extension of the anterior deprogrammer may require some time for adaptation and may elicit the gag reflex if not properly adjusted.

No significant difference was found between the investigated methods regarding ease of instructions and pain, which signifies that reduction in muscle activity was not due to nociceptive inhibition of the orofacial muscles in response to a painful stimulus^19^.

The use of a healthy sample in this study may have been a limitation, as it does not represent the broader patient population typically encountered by dental clinicians. However, this sample was selected to ensure standardization, as using a non-healthy population could compromise the study’s internal validity due to inter-participant variability and the potential influence of confounding factors. Therefore, the authors recommend evaluating muscle activity associated with various CR locating techniques in a non-healthy sample, and more specifically comparing muscle activity between asymptomatic and symptomatic TMD patients after locating CR. Another limitation of the present study is that the accuracy of condylar position in three-dimensional space was not objectively assessed. Future studies should assess the impact of CR locating devices on three-dimensional condylar position using a condylar position indicator (CPI) articulator or jaw motion analyzers (JMAs) and explore how these changes relate to muscle activity. These tools also allow visualization of CR–MIP discrepancies, which may influence muscle activity measurements and, if large or left unaddressed by the clinician, could compromise diagnostic accuracy during occlusal evaluation^50,51^. Furthermore, despite efforts to standardize conditions, variations in emotional status on different days may have influenced resting muscle activity. Future studies could incorporate measures to monitor or control participants’ emotional status, such as standardized mood assessments or stress questionnaires, to minimize their potential influence on resting muscle activity. Lastly, the CR locating techniques used in the present study were limited by their indication for dentate patients only, which also shaped the eligibility criteria for the study participants. Future studies should examine muscle activity using alternative CR locating methods that are not restricted to dentate patients.

## Conclusions

Within the limitations of this current cross-over clinical study, it was concluded that:

1. The anterior deprogrammer and Lucia Jig proved effective in reducing muscle activity while locating centric relation compared to the leaf gauge.
2. The Lucia jig, being both simple and comfortable for the patient, is recommended for locating centric relation in healthy adults who do not require long-term muscle deprogramming.

## Data Availability

The datasets generated and analyzed during the current study are available from Reham Said Elbasty (corresponding author) on reasonable request.
